# The Week's Work

**Published:** 1911-01-14

**Authors:** 


### The Week's Work.

### THE PRESBYTERIAN "DEAL."

Elsewhere in this issue ("Columbia University and the Presbyterian Hospital," page 483) we publish details of the important scheme of amalgamation which has just been entered into between the Presbyterian Hospital of New York and the faculty of medicine of the University of Columbia. By this alliance the City of New York secures a combination which is almost ideal—a model, up-to-date hospital, magnificently equipped, staffed, and managed, and a medical school which is likely to become one of the finest in America, and so rival that of the now famous Johns Hopkins. We are in doubt which to admire most—the smoothness and tact with which the negotiations which led to such an admirable result were conducted, or the munificent generosity which enabled the ideal that the authorities on both sides had in view to be achieved, as it promises to be achieved, within a short space of time. All are examples to us over here. We do not think that such a far-reaching and memorable result could have been accomplished without a certain amount of give-and-take on both sides: mutual concessions and mutual admissions, although we are not told about them, there must have been. It is just this tactful broad-mindedness that is a good augury for the future relations between the school and the hospital. In his letter to the hospital, announcing the donation of a million and a half dollars to be specially set aside for the school, Mr. Harkness pointed out that his interest was equally with the sick and with the well, but that he was more keenly concerned about the endowment of hospital research work, as distinct from general medical research. This is a concern that hospital friends might well cultivate on this side of the Atlantic. By the provision of funds to found special departments, by the equipment of such departments, and by enabling hospitals to put at the head of such specialties whole-time men who can devote themselves energetically to the work they have to do, we fancy more will be done in the future to promote knowledge and stimulate interest in science than by the endowment of special research scholarships. We cordially congratulate the Presbyterian Hospital and the Columbia University on the new coalition, which, we feel sure, will mark an epoch in American institutional philanthropy that is likely to be memorable on account of the benefits it guarantees to the community.

### MEDICAL SCHOOLS AND HOSPITALS.

Thoughtful friends of hospitals and of medicine will note with special gratification the fact that the treaty of alliance between the Presbyterian and Columbia contains a definite clause to the effect that no particle of financial support contributed by hospital subscribers shall be deviated to the funds of the school. That is a principle which we are fully prepared to welcome, and it is an encouraging sign of the broadmindedness and logical ability of those responsible for the negotiations that led to such a praiseworthy condition being proposed in the first place and honoured in the second. The King's Fund in this country has now definitely pronounced its opinion on the subject of medical schools and hospital funds, and we need not refer at length to the whole question, which we have discussed on various occasions, and more particularly just lately (see leading article, Medical Schools and the King's Fund, December 17). With the opinion laid down in the Fry Commission's Report, most hospital workers, and, we fancy, most medical men who take a calm and unprejudiced view of the facts, will be in agreement. That some of the profession do not agree we are unfortunately fully aware; the long and somewhat rambling letter of F.R.C.S. in the *Times* of Thursday, January 5, proves this. The writer, whose identity it is easy to guess, deals with exactly the points which the authorities of the Presbyterian Hospital and the Columbia University have had to consider, and the fact that the alliance between hospital and medical school has been ratified, notwithstanding a studied insistence on the *noli tangere* clause as regards the institutional funds, is in effect, an answer to F.R.C.S.'s argument, or so much of his letter as can be styled argument. But the fact that so generally admired an authority as this gentleman has thought it necessary to attack the conclusions of the Fry Commission is a sign that there still exists an amount of prejudice among medical men who are connected with medical schools with regard to this apportioning of funds.

### A RECORD OF PROGRESS AT CHESTER.

Readers of The Hospital will not have failed to notice the importance of the brief announcement, which we published last week on page 446, of the proposed reconstruction of Chester Infirmary. That proposal, and the instructive promise of £1,000 "provided the work is begun at once," completes a register of development at Chester which the files of The Hospital record. In the autumn of 1909 Sir Henry Burdett visited the infirmary and wrote in the visitors' book an account of its condition, recommending a rebuilding scheme. The cost at first sight frightened the local authorities. Nevertheless, on February 5, 1910, we were able to publish (on page 549) a note on the annual meeting of the infirmary, at which the Mayor of Chester, Mr. Alderman D. L. Hewitt, with great public spirit confessed that "the medical men who have read Sir Henry's comments in the visitors' book remark that many of them, unfortunately, are true." Three weeks later, on February 26, page 637, Mr. Alderman Hewitt is found continuing his policy by recommending courageous action to the Governors, for some of them feared that not less than £60,000 would be required for the reconstruction of the hospital. Last summer the city of Chester became the centre of public interest on account of its Pageant, the success of which was so great that a good balance remained in the hands of the Pageant Committee. On the Mayor's suggestion, part of this sum was made the nucleus of a fund " for alteration, rebuilding, or the erection of a new hospital." On August 27 we commended this suggestion to the public of Chester, and it was very gratifying to be able to report that Mr. Hewitt's proposal had been taken up locally. No doubt, too, the association of the reconstruction of the infirmary with the name of the late King has attracted many supporters. It is also satisfactory to find that the scheme is expected to cost, not £60,000, but £25,000, or, in other words, the sum estimated in our columns of February 5 last. The ultimate determination of the hospital authorities and of the people of Chester to face the condition of affairs and to have an up-to-date infirmary worthy of the city is statesmanlike and certain of support. We may note, too, that the preliminary heart-searchings and momentary reluctance to undertake the new responsibility are not the characteristics of any one city, but are to be found wherever human nature is called upon to remedy a defect that time has made habitual. Other hospitals should be encouraged by Chester's example to realise that difficulties are never so great as our first want of energy is inclined to make them.

### HOSPITALS AND THE LAW.

During the past year several interesting legal decisions affecting voluntary hospitals have been given in the English and American Courts. Some of the points raised have been of more than local importance, and we hope to deal with these *in extenso* at a later date. In one (American) case the question of a patient's consent to operation was discussed and decided; in another the matter of the right of hospitals to quarter arms and to keep uniformed male officials was thrashed out; while in a third a very important matter with reference to the title of the institution was decided. Among other interesting cases during the year we may mention the action against the Bolingbroke Hospital, and the case of the Pendlebury Children's Hospital in regard to damage done to the buildings by undermining of adjacent territory. Some useful and informative papers on the legal aspects of hospital work have been published during the year, the most recent being Goodnow and Lodge's paper on " Hospital Accidents," which we intend to discuss at length in a future issue.

### SCOTLAND AND THE MIDWIVES ACT.

In an address delivered to the Edinburgh Obstetrical Society, Dr. F. W. N. Haultain has recently expressed some interesting views about the extension of the Midwives Act—which at present applies to England only—to Scotland. An active campaign to secure this object has already commenced, and it is gratifying to learn that one reason why Scotland is anxious to adopt the Act is that the certificate of the Central Midwives Board is becoming so esteemed that the majority of women trained in Scottish maternity institutions seek it as a hall-mark of efficiency. For this they have, of course, to come south over the border to be examined, and the Scottish obstetricians are coming to the conclusion that trouble and expense might be saved could the examinations be conducted within the borders of their own country. Furthermore, Dr. Haultain says that endeavours should be directed to a special Act in which certain requirements of the English Act are modified, for they have proved irksome and unnecessary. The requirements of the Board are so exacting that it is impossible for nurses and students to be trained on the same cases; this seriously handicaps the training of both in Edinburgh and other Scottish schools, from want of sufficient clinical material. To allow of more use being made of this material and to obviate unnecessary trouble on the part of Scottish nurses who desire this certificate of efficiency Dr. Haultain is in favour of a Scottish diploma to be granted on lines similar to those of the English model, but modified in these respects. The position, he adds, is of extreme importance, and must be at once energetically faced.

### THIS WEEK'S DRUG MARKET.

Business in the drug market has now assumed a more normal appearance, and there is a fair amount of inquiry, which may lead to orders. As was expected, the price of santonin has been advanced 2s. 3d. per lb., otherwise there have been few definite changes in value. Saffron still shows a tendency to advance in price, and sugar of milk seems likely to increase in value to a slight extent. Oil of cubebs is cheaper. The value of almond oil is well maintained. Camphor seems more likely to advance in price than to recede. Ergot of rye continues to fetch high prices. Atropine is slightly lower in value.

